# Global analysis of birth statistics from civil registration and vital statistics systems

**DOI:** 10.2471/BLT.22.289035

**Published:** 2023-11-02

**Authors:** Tim Adair, Azza Badr, Lene Mikkelsen, Jessica Hooper, Alan D Lopez

**Affiliations:** a The Nossal Institute for Global Health, Melbourne School of Population and Global Health, University of Melbourne, 32 Lincoln Square North, Carlton 3053, Victoria, Australia.; b Division of Data, Analytics and Delivery for Impact, World Health Organization, Geneva, Switzerland.; c LM Consulting, Tamborine Mountain, Queensland, Australia.; d Manchester, England.

## Abstract

**Objective:**

To assess civil registration and vital statistics completeness for births in World Health Organization’s Member States and identify data completeness gaps.

**Methods:**

For the 194 Member States, we sourced birth registration data from the United Nations Children’s Fund database of national surveys, and, where available, vital registration reports. We acquired publicly available vital statistics compiled by national authorities. We determined civil registration completeness as the percentage of living children younger than five years whose births have been reported as registered. We evaluated vital statistics completeness against the United Nations World Population Prospects' live birth estimates, and grouped countries into seven categories based on their civil registration and vital statistics completeness.

**Findings:**

Globally, civil registration completeness for births was 77%, exceeding vital statistics completeness for births at 63%. Twenty countries had limited civil registration (25% to 74% completeness) and had nascent or no vital statistics data (completeness < 25%) for births. Five countries had nascent or no civil registration and vital statistics for births. Twenty countries had functional civil registration (75% to 94% completeness) but nascent or no available vital statistics. Approximately half (96) of the countries had complete civil registration and vital statistics for births, but contributed to only 22% of global births.

**Conclusion:**

The gap in completeness between civil registration data and vital statistics for births is most pronounced in countries with lower civil registration completeness. Enhancing data transfer processes for birth registration, along with targeted investments to elevate registration rates, is crucial for yielding comprehensive fertility statistics for governmental planning.

## Résumé

**Objectif:**

Évaluer le degré d'exhaustivité de l'enregistrement des faits d'état civil et de statistiques d'état civil pour les naissances au sein des États Membres de l'Organisation mondiale de la Santé et identifier les lacunes dans les données.

**Méthodes:**

Pour les 194 États Membres, nous nous sommes procuré les informations relatives à l'enregistrement des naissances dans la base de données sur les enquêtes nationales du Fonds des Nations Unies pour l'enfance et, le cas échéant, dans les rapports d'enregistrement des faits d'état civil. Nous avons également récupéré les statistiques d'état civil accessibles au public et recueillies par les autorités nationales. Nous avons ensuite mesuré l'exhaustivité des faits d'état civil en calculant le pourcentage d'enfants vivants de moins de cinq ans dont la naissance a été indiquée comme déclarée. Enfin, nous avons évalué l'exhaustivité des statistiques d'état civil en fonction des naissances vivantes estimées par les Nations Unies dans leurs Perspectives de la population mondiale, avant de classer les pays en sept catégories selon leur exhaustivité en la matière.

**Résultats:**

À l'échelle mondiale, l'exhaustivité des faits d'état civil pour les naissances s'élevait à 77%, dépassant ainsi les 63% obtenus pour l'exhaustivité des statistiques d'état civil. Dans vingt pays, l'enregistrement des faits d'état civil était limité (25% à 74% d'exhaustivité) et les données concernant les statistiques d'état civil étaient soit rudimentaires, soit inexistantes (exhaustivité < 25%) pour les naissances. Cinq pays disposaient de systèmes d'enregistrement des faits d'état civil et de statistiques d'état civil rudimentaires ou inexistants pour les naissances. Vingt possédaient un système d'enregistrement des faits d'état civil fonctionnel (75% à 94% d'exhaustivité) mais des statistiques d'état civil rudimentaires ou inexistantes. Et près de la moitié (96) des pays étaient munis d'un système complet d'enregistrement des faits d'état civil et de statistiques d'état civil, mais ne représentaient que 22% des naissances à travers le monde.

**Conclusion:**

En ce qui concerne les naissances, les différences d'exhaustivité entre les données d'enregistrement des faits d'état civil et les statistiques d'état civil sont principalement observées dans les pays où les données sur les faits d'état civil comportent des lacunes. Afin de fournir des statistiques détaillées sur la fertilité, utiles à la planification gouvernementale, il est essentiel d'améliorer les procédures de transfert des données pour l'enregistrement des naissances, mais aussi d'investir de manière ciblée pour faire progresser les taux d'enregistrement.

## Resumen

**Objetivo:**

Evaluar la completitud del registro civil y las estadísticas vitales de los nacimientos en los Estados Miembros de la Organización Mundial de la Salud e identificar lagunas en la completitud de los datos.

**Métodos:**

Para los 194 Estados Miembros, se obtuvieron los datos de registro de nacimientos de la base de datos de encuestas nacionales del Fondo de las Naciones Unidas para la Infancia y, cuando estaban disponibles, de los informes de registro civil. Además, se obtuvieron las estadísticas vitales de acceso público que recopilan las autoridades nacionales. Se determinó la completitud del registro civil como el porcentaje de niños vivos menores de cinco años cuyos nacimientos se habían notificado como registrados. Se evaluó la completitud de las estadísticas vitales comparándola con las estimaciones de nacidos vivos de las Perspectivas de la Población Mundial de las Naciones Unidas y se agruparon los países en siete categorías según la completitud del registro civil y de las estadísticas vitales.

**Resultados:**

En todo el mundo, la completitud del registro civil para los nacimientos era del 77%, superando la completitud de las estadísticas vitales para los nacimientos en un 63%. Veinte países tenían un registro civil limitado (entre un 25% y un 74% de completitud) y datos incipientes o inexistentes de estadísticas vitales (completitud <25%) para los nacimientos. Cinco países carecían de registro civil y de estadísticas vitales para los nacimientos. Veinte países contaban con un registro civil funcional (entre el 75% y el 94% de completitud), pero con estadísticas vitales incipientes o inexistentes. Aproximadamente la mitad (96) de los países disponían de registro civil y estadísticas vitales completos para los nacimientos, pero solo representaban el 22% de los nacimientos mundiales.

**Conclusión:**

La diferencia de completitud entre los datos del registro civil y las estadísticas vitales para los nacimientos es más evidente en los países con un registro civil menos completo. La mejora de los procesos de transferencia de datos para el registro de nacimientos, junto con inversiones específicas para elevar las tasas de registro, es esencial para obtener estadísticas de fecundidad completas que faciliten la planificación gubernamental.

## ملخص

**الغرض:**

تقييم مدى اكتمال التسجيل المدني والإحصاءات الحيوية للمواليد في الدول الأعضاء في منظمة الصحة العالمية وتحديد الفجوات في اكتمال البيانات.

**الطريقة:**

بالنسبة للدول الأعضاء البالغ عددها 194 دولة، حصلنا على بيانات تسجيل المواليد من قاعدة بيانات المسوحات الوطنية لصندوق الطفولة التابع للأمم المتحدة، وتقارير التسجيل الحيوية حيثما كانت متاحة. حصلنا على إحصاءات حيوية متاحة للجمهور، وقامت بتجميعها السلطات الوطنية. لقد حددنا مدى اكتمال التسجيل المدني كنسبة مئوية من الأطفال الأحياء الذين تقل أعمارهم عن خمس سنوات، والذين تم الإبلاغ عن ولادتهم على أنها مسجلة. وقمنا بتقييم مدى اكتمال الإحصاءات الحيوية في مقابل تقديرات المواليد الأحياء الصادرة عن التوقعات السكانية العالمية للأمم المتحدة، وقمنا بتجميع الدول في سبع فئات بناءً على تسجيلها المدني واكتمال إحصاءاتها الحيوية.

**النتائج:**

على الصعيد العالمي، بلغت نسبة اكتمال التسجيل المدني للمواليد %77، متجاوزة بذلك نسبة اكتمال الإحصاءات الحيوية للمواليد التي بلغت %63. كان التسجيل المدني محدودًا في عشرين دولة (تراوحت نسبة الاكتمال بين %25 و%74)، وكانت لديها بيانات إحصائية حديثة العهد أو معدومة (نسبة الاكتمال أقل من %25) للمواليد. وكانت خمس دول لديها تسجيل مدني حديث العهد أو معدوم للتسجيل المدني والإحصاءات الحيوية للمواليد. وكانت عشرون دولةلديها تسجيل مدني فعال (اكتمال بنسبة %75 إلى %94)، ولكن لا توجد إحصاءات حيوية متاحة أو كانت حديثة العهد. كان لدى ما يقرب من نصف الدول (96) تسجيل مدني كامل وإحصاءات حيوية للمواليد، لكنها ساهمت بنسبة %22 فقط من حالات الولادة العالمية.

**الاستنتاج:**

إن الفجوة في الاكتمال بين بياناتالتسجيل المدني والإحصاءات الحيوية للمواليد تكون أكثر وضوحا في الدول التي تكون فيها نسبة اكتمال التسجيل المدني أقل. إن تعزيز عمليات نقل البيانات لتسجيل المواليد، إلى جانب الاستثمارات المستهدفة لرفع معدلات التسجيل، هو أمر حيوي للحصول على إحصاءات شاملة للخصوبة لأغراض التخطيط الحكومي.

## 摘要

**目的:**

评估世界卫生组织成员国户籍登记和人口统计数据中出生登记数据的完整性，并确定数据完整度方面的差距。

**方法:**

针对 194 个成员国，我们从联合国儿童基金会的国家调查数据库中获取了出生登记数据，同时，还获取了主要的人口登记报告（如有）。我们获取了国家权威部门编制的公开的人口统计数据。我们将户籍登记的完整性定义为五岁以下存活儿童中占出生时已登记儿童的百分比。我们根据联合国世界人口展望估算的活产婴儿数对人口统计数据的完整性进行了评估，并根据其户籍登记和人口统计数据的完整性将国家分为七类。

**结果:**

在全球范围内，户籍登记中出生数据的完整度为 77%，超过了出生人口数据统计的 63%。20 个国家的户籍登记数据非常有限（完整度为 25% 至 74%），并且刚开始进行出生人口数据统计或没有出生人口数据统计（完整度低于 25%）。5 个国家刚开始进行或没有户籍登记和出生人口数据统计。20 个国家有实用的户籍登记数据（完整度为 75% 至 94%），但没有初步的或可用的人口统计数据。大约一半（96 个）国家拥有完整的出生户籍登记和人口统计数据，但完整度仅占全球出生人数的 22%。

**结论:**

在户籍登记数据完整度较低的国家，户籍登记数据与出生人口统计数据之间的完整度差距最为明显。完善出生登记数据的传输过程，同时进行有针对性的投资以提高登记率，对于为政府规划提供全面的生育统计数据至关重要。

## Резюме

**Цель:**

Оценить полноту данных регистрации актов гражданского состояния и учета естественного движения населения о рождаемости в государствах-членах Всемирной организации здравоохранения и выявить пробелы в полноте данных.

**Методы:**

Для 194 государств-членов были использованы данные о регистрации рождаемости из базы данных Детского фонда ООН по национальным опросам, а также при наличии отчеты о регистрации актов гражданского состояния. Были получены общедоступные статистические данные о естественном движении населения, собранные национальными органами власти. Полноту регистрации актов гражданского состояния определяли как долю живых детей в возрасте до пяти лет, рождение которых было зарегистрировано. Была проведена оценка полноты статистики естественного движения населения в сравнении с оценками живорождения, полученными в рамках программы ООН «Перспективы мирового народонаселения». Страны были разделены на семь категорий в зависимости от полноты регистрации актов гражданского состояния и статистики естественного движения населения.

**Результаты:**

В целом по миру полнота регистрации актов гражданского состояния при рождении составила 77%, превысив полноту естественного движения населения при рождении на 63%. В двадцати странах регистрация актов гражданского состояния была ограниченной (полнота от 25 до 74%), а данные статистики естественного движения населения (полнота < 25%) по рождаемости находятся в стадии становления или вовсе отсутствуют. В пяти странах регистрация актов гражданского состояния и статистика естественного движения населения по рождаемости находятся в стадии становления или вовсе отсутствуют. В двадцати странах регистрация актов гражданского состояния была функциональной (полнота от 75 до 94%), но статистика естественного движения населения находилась в стадии становления или вовсе отсутствовала.. Примерно у половины (96) стран наблюдается полная регистрация актов гражданского состояния и статистика естественного движения населения при рождении, однако на их долю приходится лишь 22% от общего числа случаев рождения в мире.

**Вывод:**

Разрыв в полноте данных регистрации актов гражданского состояния и статистики естественного движения населения в отношении случаев рождения наиболее выражен в странах с более низкой полнотой регистрации актов гражданского состояния. Усовершенствование процессов передачи данных для регистрации случаев рождения, а также целевые инвестиции для повышения уровня регистрации имеют решающее значение для получения полной статистики рождаемости в целях планирования на государственном уровне.

## Introduction

Evaluations of the performance of civil registration and vital statistics systems should emphasize both the functions of legal registration and certification of vital events, and the production of vital statistics from these data.^1^ For births, the civil registration component is particularly important because it provides several benefits to both individuals and families such as proof of age, citizenship, and access to government social services (education and health care).^2^^–^^4^ A major objective of governments and donors such as the United Nations Children’s Fund (UNICEF) is to increase the percentage of children younger than five years whose birth is registered. Increasing birth registration is the primary indicator for sustainable development goal (SDG) 16.9, which aims to achieve legal identity for all by 2030, including birth registration.^5^ Recent efforts to strengthen civil registration and vital statistics systems in relation to birth registration have mainly focused on improving the civil registration of births and issuance of birth certificates.

While the focus on civil registration of births has undoubtedly brought benefits to individuals and families, the role of civil registration and vital statistics systems in producing routine fertility statistics has received considerably less attention. High-quality birth registration data provide timely and accurate data on fertility at the national and subnational level, and can be used to generate key indicators such as the total fertility rate and age-specific fertility rates (according to age of the mother). Governments can use these data to understand fertility trends and variations within their population, and use these data as inputs into population projections for the planning of health, education and other government services.^6^ Fertility data can also be the denominator for early age mortality indicators, which are important indicators of population health. The use of civil registration and vital statistics data to measure fertility overcomes several limitations of survey and census-derived estimates such as use of retrospective data, irregular collection and sampling uncertainty.

In earlier worldwide evaluations of the comprehensiveness of birth registrations in civil records, as presented in the UNICEF *State of the world's children 2019* report, estimations were based on survey responses from households. These estimations indicated that 73% of children younger than five years were reported to have their births registered.^7^ Previously, the most comprehensive previous assessment of vital statistics for births estimated that only 40% of global births in 2011 were registered (the year with the highest completeness).^8^ The substantial difference between these figures likely arises due to: (i) different reference dates; (ii) the self-reported nature of survey birth registration data (which may overstate completeness); and (iii) that civil registration is a requirement for subsequent production of vital statistics.^9^

Here we provide an updated assessment and comparison of civil registration and vital statistics systems in each country and World Health Organization (WHO) region in terms of the completeness of the civil registration of births and vital statistics that are produced from these data. We categorize countries into seven groups by considering both their performance in civil registration and vital statistics concerning births. This classification is based on an assessment of birth statistics completeness generated by civil registration and vital statistics systems. Additionally, we incorporate existing survey-based estimates of birth registration completeness among children younger than five years. We conduct an analysis of all seven categories in each WHO region and globally. Our findings are intended to serve as a basis for guiding interventions aimed at enhancing the registration of births, and the subsequent compilation and utilization of data as routine fertility statistics.

## Methods

### Data source

To measure the completeness of both civil registration of births and vital statistics of births, we used two distinct data sources. We obtained data on the birth registration status of children younger than five years at household level from a UNICEF database, which collates information from Demographic and Health Surveys (DHS) and Multiple Indicator Cluster Surveys (MICS), from other national surveys or vital registration reports for countries with complete birth registration.^10^^–^^12^ In these surveys, respondents are asked whether the child has a birth certificate and, if not, whether or not the birth is registered with a civil authority. To ensure inclusion of as many countries as possible, we used the surveys conducted from 2011 to 2019. 

We obtained data for vital statistics of births from routine government registration or reporting systems with the goal of counting all events within their jurisdiction which are, or should be, integrated into the national civil registration and vital statistics system. We searched for birth data that national authorities compiled and made publicly available. These sources include: (i) national reports; (ii) United Nations (UN) Sustainable Development *Population and vital statistics* Report;^13^ (iii) Global Burden of Disease (GBD) Live Births Database;^14^ (iv) civil registration and vital statistics assessments; and (v) personal communications. All vital statistics data sources are available in the online repository.^15^

### Analysis

#### Civil registration completeness

We measured completeness of civil registration data for births as the percentage of living children younger than five years whose birth is reported as being registered; consistent with the indicator for SDG 16.9 and with statistics produced in *State of the world’s children*.^5^^,^^7^

#### Vital statistics completeness 

We base our estimates of vital statistics completeness on such data exclusively from 2015–2019 because of the importance of timeliness for the production of vital statistics. We also present vital statistics completeness estimates in bands of five percentage points to account for general uncertainty in our estimated total births.

For this analysis, vital statistics completeness was calculated as registered or reported births divided by United Nations World Population Prospects’ estimate of live births for that year.^16^

We excluded still births, and measured completeness by year of birth occurrence and for births registered within one year of occurrence where possible, to avoid biasing the data due to late registrations. For countries where data were only available by year of registration, if there was minimal change (in either direction) in annual registered births in recent years, then we used the most recent year of data. This implied that there would not be bias in the data from late registrations, and that the annual number of registered births would approximately equal the annual number of birth occurrences. If there was moderate change (i.e. 10%–25%) in annual registered births in recent years, we used an average of the number of registered births over recent years. If there were large changes (i.e. more than 25%) in the number of registered births per annum or implausibly high levels of completeness (e.g. over 100%), we chose not to use the country’s data. Both the UN and GBD estimate live births in countries with incomplete birth registration mostly from census and survey data using demographic and statistical models.^16^^,^^17^ In some countries, the denominator was the GBD’s estimated births because it provided a more plausible estimate of completeness than the UN’s estimated births; criteria for choosing the GBD rather than UN-estimated births can be found in the online repository.^15^^,^^17^ In other countries, we used the completeness estimate in the WHO SCORE Global Report if it were more plausible than that made using numerator data and the UN or GBD’s estimate of total births, or if numerator data could not be obtained.^18^

### Classification

We classified countries into seven categories based on our estimates of civil registration and vital statistics completeness. For our purposes, civil registration completeness includes births registered after one year and before five years, and uses a broader time period than vital statistics completeness (2011–2019 compared with 2015–2019) because timeliness is less important for civil registration than vital statistics. These distinctions are important to keep in mind when interpreting the results. 

We defined seven categories based on data utility (Table 1). Countries with vital statistics completeness of 95% and above are classified as complete civil registration and vital statistics; and those with vital statistics completeness of 75%–94% are classified as functional civil registration and vital statistics (that is, the system is clearly functional but could not be regarded as complete). We do not separately classify civil registration completeness because it is assumed to be at least as high as vital statistics completeness (that is, the former is a requirement for the latter). Where either civil registration or vital statistics completeness is between 25%–74%, we classified it as limited civil registration or vital statistics, and as nascent or non-existent if below 25% or no data are available. We present findings as a percentage of estimated total births (estimates from the UN World Population Prospects) across all seven categories.^16^ Finally, we also calculated regional and global civil registration and vital statistics completeness.

**Table 1:** Classification of countries by civil registration and vital statistics completeness or births

Categories	Completeness, %	
	Civil registration	Vital statistics
Complete civil registration and vital statistics	NA	≥ 95
Functional civil registration and vital statistics	NA	75–94
Functional civil registration, limited vital statistics	≥ 75	25–74
Functional civil registration, nascent or no vital statistics	≥ 75	< 25
Limited civil registration, limited vital statistics	25–74	25–74
Limited civil registration, nascent or no vital statistics	25–74	< 25
Nascent or no civil registration, nascent or no vital statistics	< 25	< 25

NA: not applicable.

Note: Countries with no data were categorized as having less than < 25% completeness data.

## Results

**Fig. 1:**
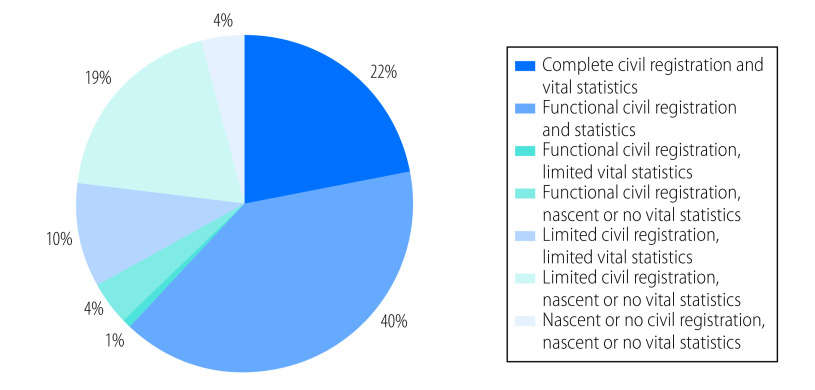
Percentage of estimated global births in each category of civil registration and vital statistics; 2019

Note: The different categories are defined in Table 1.

Out of the 194 WHO Member States, 96 (49%) are classified as having complete civil registration and vital statistics systems for births (Table 2). However, these countries only comprise an estimated 22% of global births, because of the low number of births relative to population in these countries (Fig. 1). A further 37 countries comprising an estimated 40% of global births have functional civil registration and vital statistics systems for births; and only five countries have functional civil registration but limited or nascent/no vital statistics. Eleven countries have limited civil registration and limited vital statistics; a further 20 countries have limited civil registration and nascent or no vital statistics; and five countries have nascent or no civil registration and vital statistics. Overall, global civil registration completeness (77%) is higher than vital statistics completeness (63%).

**Table 2:** Percentage of estimated births in each civil registration and vital statistics category, 2019

Variable	No. of countries	Estimated % of births						
		WHO region						Global
		African	Americas	South-East Asia	European	Eastern Mediterranean	Western Pacific	
**Category**								
Complete civil registration and vital statistics	96	3	78	3	76	31	17	22
Functional civil registration and vital statistics	37	10	19	72	23	12	80	40
Functional civil registration, limited vital statistics	5	1	1	0	0	0	2	1
Functional civil registration, nascent or no vital statistics	20	9	2	3	1	6	0	4
Limited civil registration, limited vital statistics	11	11	0	8	0	40	2	10
Limited civil registration, nascent or no vital statistics	20	54	0	14	0	8	0	19
Nascent or no civil registration, nascent or no vital statistics	5	11	0	0	0	4	0	4
Total	194	100	100	100	100	100	100	100
**Completeness**								
Civil registration, 2011–2019^a^	NA	49	97	88	97	69	90	77
Vital statistics, 2015–2019	NA	20	95	73	97	52	90	63
**Estimated births of total global births**	NA	27	11	25	8	13	17	100

NA: not applicable; WHO: World Health Organization.

^a^ Calculated using vital statistics completeness for countries with complete or functional civil registration and vital statistics systems.

In the African Region, which contains the highest proportion of global births of any region (27%), over half (54%) of its births are in countries with limited civil registration and nascent or no vital statistics, while 11% are in countries with nascent or no civil registration and vital statistics for births. The poor condition of both civil registration and especially vital statistics for births is shown by only 3% of births being in countries with complete civil registration and vital statistics, and 10% with functional civil registration and vital statistics for births. These findings result in civil registration completeness in the African Region of 49% but vital statistics completeness of just 20% (Table 2).

The Eastern Mediterranean Region has overall higher levels of registration (69%) than vital statistics completeness (52%). Forty per cent of the births in the region are in countries with limited civil registration and limited vital statistics for births, and a further 12% with limited or nascent/no civil registration and nascent/no vital statistics for births. 

In the South-East Asia Region, where one-quarter of the world’s births occur, 72% of births occur in countries (predominantly India) with functional civil registration and vital statistics for births. However, 22% of births in that region have limited civil registration and either limited or nascent/no vital statistics, resulting in civil registration completeness of 88% and vital statistics completeness of 73%. The Region of the Americas, European and Western Pacific Regions each have relatively high vital statistics completeness of at least 90%. However, a few of the smaller Western Pacific countries have limited civil registration while China, which comprises most births in this region, has a functional civil registration and vital statistics system for births. Table 2 presents the regional disaggregation according to the number of countries in each category.

**Fig. 2:**
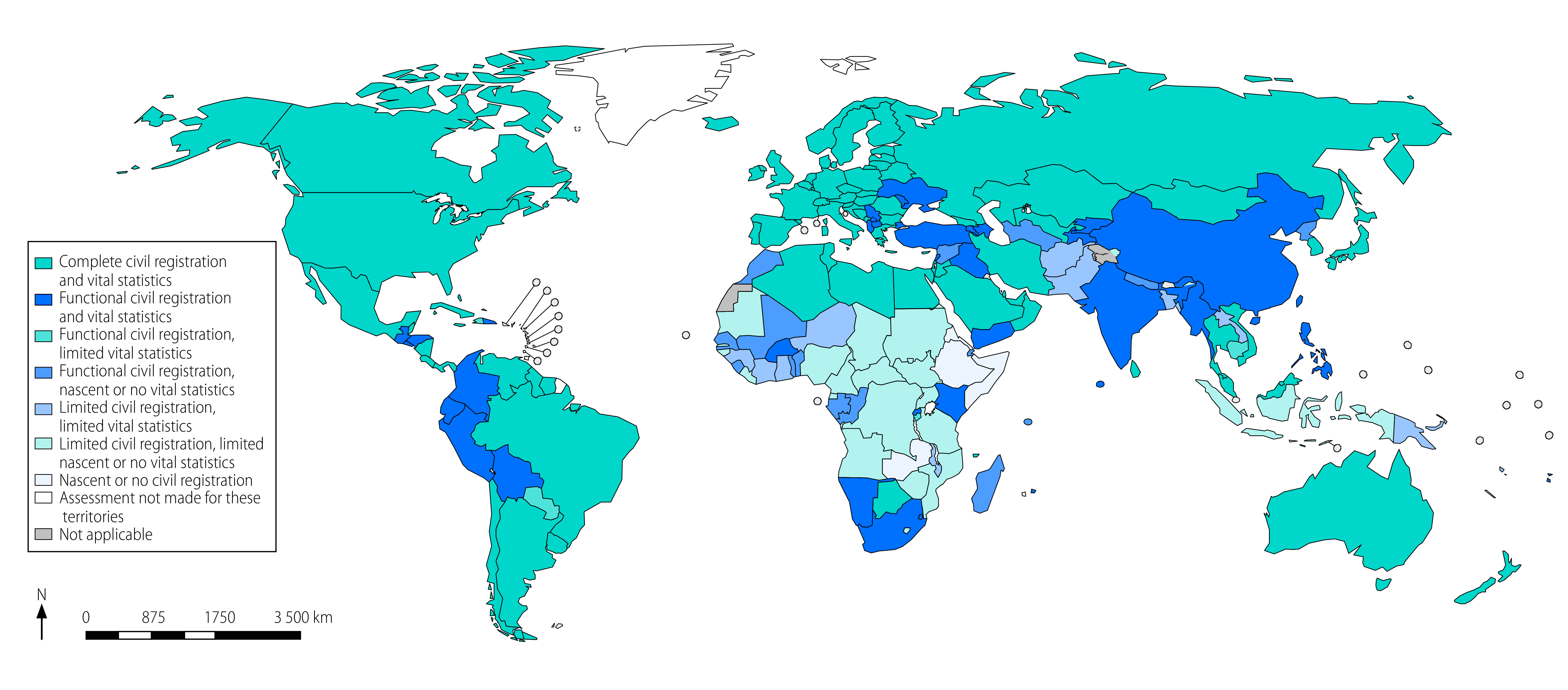
Completeness of civil registration and vital statistics for births in WHO Member States

A notable finding for Africa is that nine countries have functional civil registration for births, with at least 75% of children younger than five years reported to have had their birth registered, but no vital statistics: Benin, Comoros, Congo, Gabon, Madagascar, Mali, Senegal, Sierra Leone and Togo (Fig. 2). A further 17 countries have limited civil registration but nascent or no vital statistics, or have no civil registration nor vital statistics, including countries with large populations such as Cameroon, Democratic Republic of the Congo, Eritrea, Ethiopia, Mozambique, Nigeria and Zambia (online repository).^15^

WHO: World Health Organization.

Note: The different categories are defined in Table 1.

In the Eastern Mediterranean Region, civil registration completeness and vital statistics completeness in two large countries (Afghanistan and Pakistan) is less than 50%; while Sudan has limited civil registration and no vital statistics; and Somalia has no civil registration nor vital statistics (online repository).^15^

In the South-East Asia Region, Nepal has functional civil registration (77%) but no vital statistics; Indonesia and Timor-Leste have limited civil registration and no vital statistics for births (online repository).^15^

In the Western Pacific Region, Papua New Guinea and Vanuatu have limited civil registration and vital statistics for births (online repository).^15^

## Discussion

We found that just over three quarters of global births are registered while data for just over three fifths are available as vital statistics. While almost half of WHO Member States have complete civil registration and vital statistics systems for births, these countries have a low proportion of the total global births because they are predominantly high-income countries with low birth rates. Our results indicate that low vital statistics completeness for births, either with functional, limited, nascent or no civil registration for births, are mainly seen in populous countries and countries with a high fertility rate. These disparities lower global vital statistics completeness estimates^16^ because these countries account for a substantial portion of the estimated global births. 

The completeness for vital birth statistics of 63% is lower than the 70% completeness of death statistics from civil registration and vital statistics systems.^19^ This difference is attributable to countries that are less likely to record vital statistics, that have relatively young populations, experience high birth rates and have lower crude death rates. 

A notable finding of this study is that, in most instances, the gap between civil registration and vital statistics completeness is greater in countries with lower civil registration completeness, particularly in the African and Eastern Mediterranean regions. That is, in poorer-performing systems the production and publication of vital statistics is very low or non-existent, in contrast to better-performing systems where all or almost all registered births are published as vital statistics. This result may be because civil registration and vital statistics procedures in countries with less mature civil registration and vital statistics systems are poorly integrated with each other due to insufficient coordination between system stakeholders. Such countries have understandably focused on improving the civil registration of births, but have not invested in producing vital statistics from these data. Where birth registration is known to be less than complete, Member States may instead rely on household-level surveys and censuses to collect fertility data. When vital statistics data are not readily available or usable, policy-makers might not understand their importance and not recognize the need to invest in the vital statistics component of the system. Additionally, high levels of civil registration completeness in some countries could lead to misplaced confidence in the performance of the civil registration and vital statistics system, even though one of its primary functions (vital statistics) is not being performed. In contrast, civil registration and vital statistics systems that are more robust, better organized, and have access to sufficient resources are better positioned to generate and release vital statistics derived from such data.

In all countries, even those with incomplete registration of births, there need to be improved processes by which data are transferred to a relevant authority (e.g. national statistical office) who can then assess their completeness and quality, and analyse and disseminate them as fertility statistics disaggregated by age of mother and sex of child.^20^^,^^21^ This intervention may require mapping^22^ of the business processes of the current civil registration and vital statistics system to identify inefficiencies in the generation of vital statistics. A substantial number of countries without available routine birth statistics, but with significant proportions of children with registered births, suggest that such mapping would be beneficial to improve their vital statistics system. 

Continued implementation of effective interventions is required to improve the percentage of children whose birth is registered; particularly in the African Region where a major percentage of global births are projected to occur in the future.^16^ Actions such as legal reforms, removal of fees for birth registration, and awareness-raising campaigns have all increased the completeness of birth registration in countries such as Brazil, Senegal and Viet Nam.^23^ Financial incentives can also be effective at increasing birth registration, with the caveat that these incentives may not result in timely birth registration, as has happened in Nepal, and may be difficult for governments to maintain.^24^^,^^25^

There can also be improvements in civil registration and vital statistics systems by using existing births data collected in health facilities or through exploiting the knowledge of midwives of non-facility births, removing the onus of notification off the family. The use of mobile registration agents can also help reach families in more rural or remote locations. In the United Republic of Tanzania, for example, registration via mobile phone applications has facilitated notable increases in birth notification and registration completeness.^26^

National governments should ensure that basic vital statistics data are reported (disaggregated at a minimum by age and sex, and preferably also by birth order and birth weight) in a timely manner in both national vital statistics reports and to international databases, to enable improved understanding of fertility patterns and trends.^8^ The *Civil registration and vital statistics strategic implementation plan 2021–2025* provides a framework for governments to use to improve the quality of their birth statistics. This plan emphasizes a few key factors: (i) strengthening coordination between the health sector and other national civil registration and vital statistics stakeholders; (ii) strengthening vital event notification integration with civil registration; (iii) building capacity to analyse data for policy purposes; and (iv) improving the reporting, production and dissemination of vital statistics.^27^ The analysis of self-reported birth registration data in DHS and MICS highlights weaknesses in the global procedures for civil registration of births, with many children not being counted, deprived of citizenship and other basic human rights. Generally, populations can benefit from complete and reliable vital statistics on births to inform policy and decision-making in areas such as education and health care.

The coronavirus disease 2019 (COVID-19) pandemic had a major impact on civil registration and vital statistics systems globally. The majority of countries’ civil registration and vital statistics systems were interrupted during the pandemic, which adversely affected birth registration.^28^ In particular, the pandemic exposed weaknesses in existing systems.^28^^,^^29^ Despite these setbacks, the pandemic hastened the use of online birth registration which will benefit civil registration and vital statistics systems in future.^30^^,^^31^ We have assessed birth registration and vital statistics before the pandemic to gauge performance in a period of more normal civil registration and vital statistics system operation.

While the indicator we selected to measure completeness of registration of births is consistent with previous measurement, it has limitations. First, the actual birth registration numbers may be over-reported because of concern about being penalized for non-registration of birth or because of confusion about whether registration has actually occurred.^9^ Second, data are only collected for live children; registration completeness of deceased children is likely lower than for those who survive, and may contribute to over-estimation of overall civil registration completeness. Third, this indicator measures completeness only for those children younger than five years and does not consider timing of registration. Despite these limitations, this indicator can provide a good approximation of the level of civil registration which can be used to generate fertility statistics for policy, provided their limitations are recognized.^16^ The vital statistics completeness definition is more stringent in terms of timing of data compared with civil registration completeness, but this is consistent with these components being more important for statistics. The criteria for eligible data sources for vital statistics completeness mean they are likely lower than if based on data available to national authorities. Vital statistics completeness results are also dependent upon the accuracy of total births estimates used as the denominator, although precise measures of completeness are less important than categorization of countries that can guide investments to strengthen civil registration and vital statistics systems. We were unable to assess the role of public versus private institutions in reporting births, because available information did not distinguish by whether they were reported by each type of facility.

Reliable vital statistics of births are valuable as they provide timely evidence on fertility that has several policy uses for governments, including the planning of health care, education and other government social services. Targeted investments into civil registration and vital statistics systems will further boost the utility of reliable and timely routine fertility statistics that can be generated to facilitate global health and social development.
