# Corrigendum

**DOI:** 10.2471/BLT.24.110124

**Published:** 2024-01-01

In: Adair T, Badr A, Mikkelsen L, Hooper J, Lopez AD. Global analysis of birth statistics from civil registration and vital statistics systems. Bull World Health Organ. 2023 Dec 1;101(12):768–776, 

on page 769, the title of Table 1 should read as follows:

Table 1. Classification of countries by civil registration and vital statistics completeness of births

